# A large kindred of pulmonary fibrosis associated with a novel ABCA3 gene variant

**DOI:** 10.1186/1465-9921-15-43

**Published:** 2014-04-15

**Authors:** Ilaria Campo, Michele Zorzetto, Francesca Mariani, Zamir Kadija, Patrizia Morbini, Roberto Dore, Eva Kaltenborn, Sabrina Frixel, Ralf Zarbock, Gerhard Liebisch, Jan Hegermann, Christoph Wrede, Matthias Griese, Maurizio Luisetti

**Affiliations:** 1 Pneumology Unit, IRCCS San Matteo Foundation Hospital, Piazza Golgi 1, Pavia 27100, Italy; 2 Department of Molecular Medicine, Section of Pathology, University of Pavia and Foundation IRCCS Policlinico S. Matteo, Pavia, Italy; 3 Institute of Radiology, IRCCS San Matteo Foundation Hospital, Pavia, Italy; 4 Pediatric Pneumology, Dr. von Hauner Children's Hospital, Ludwig-Maximilians University, Member of the German Center for Lung Research (DZL), Munich, Germany; 5 Institute for Clinical Chemistry and Laboratory Medicine, University of Regensburg, Regensburg, Germany; 6 Institute of Functional and Applied Anatomy, Hannover Medical School, Hannover, Germany and Biomedical Research in Endstage and Obstructive Lung Disease Hannover (BREATH), Member of the German Center for Lung Research (DZL), Hannover, Germany

**Keywords:** Surfactant system, Surfactant protein C, Familial fibrosis, Gene sequencing

## Abstract

**Background:**

Interstitial lung disease occurring in children is a condition characterized by high frequency of cases due to genetic aberrations of pulmonary surfactant homeostasis, that are also believed to be responsible of a fraction of familial pulmonary fibrosis. To our knowledge, ABCA3 gene was not previously reported as causative agent of fibrosis affecting both children and adults in the same kindred.

**Methods:**

We investigated a large kindred in which two members, a girl whose interstitial lung disease was first recognized at age of 13, and an adult, showed a diffuse pulmonary fibrosis with marked differences in terms of morphology and imaging. An additional, asymptomatic family member was detected by genetic analysis. Surfactant abnormalities were investigated at biochemical, and genetic level, as well as by cell transfection experiments.

**Results:**

Bronchoalveolar lavage fluid analysis of the patients revealed absence of surfactant protein C, whereas the gene sequence was normal. By contrast, sequence of the ABCA3 gene showed a novel homozygous G > A transition at nucleotide 2891, localized within exon 21, resulting in a glycine to aspartic acid change at codon 964. Interestingly, the lung specimens from the girl displayed a morphologic usual interstitial pneumonitis-like pattern, whereas the specimens from one of the two adult patients showed rather a non specific interstitial pneumonitis-like pattern.

**Conclusions:**

We have detected a large kindred with a novel ABCA3 mutation likely causing interstitial lung fibrosis affecting either young and adult family members. We suggest that ABCA3 gene should be considered in genetic testing in the occurrence of familial pulmonary fibrosis.

## Introduction

Pulmonary surfactant, a lipid-protein complex, is synthesized, packaged, and secreted by alveolar type II cells. The lipid portion constitutes approximately 90%, whereas the protein portion constitutes approximately 10% by weight of pulmonary surfactant. The latter is primarily composed of four surfactant-associated proteins (SP-) A, B, C and D. An important component in surfactant metabolism is the ATP binding cassette member A3 (ABCA3), a transporter protein that is thought to be involved in the inward transport of phospholipid into lamellar bodies. Approximately half of the alveolar surfactant pool is cleared through a GM-CSF-dependent alveolar macrophage pathway. Most of the remaining surfactant is then taken up by type II epithelial cells and transported to the lamellar body, while a portion is degraded in the respective lysosomes [1].

Mutations in the genes for surfactant proteins have been associated with interstitial lung diseases (ILD). Interestingly, ILD associated with mutations in SFTPC and SFTPA genes usually occur later in infancy or in adulthood, while ILD associated with mutations in SFTPB and ABCA3 genes generally consist of respiratory failure in term newborns [2,3]. Of interest, patients with rare mutations in SFTPA2 have been described in familial lung fibrosis [4].

More than 150 distinct mutations have been identified in the ABCA3gene, making this the largest class of mutations that cause genetic abnormalities in surfactant metabolism. Lung disease caused by ABCA3 mutations is an inherited autosomal recessive disorder, requiring mutations on both alleles. However, a significant fraction of reported cases are compound heterozygous resulting in variable expression in the neonatal period or in infancy [3].

The clinical spectrum and severity of lung disease caused by ABCA3 deficiency is extremely variable, strongly depending on the mutations found and the patho-morphological pattern induced. The majority of patients with ABCA3 mutations exhibit severe respiratory distress and failure in the neonatal period or in infancy [5-7]. Lamellar bodies are abnormally small and dense, with eccentrically placed inclusions creating a characteristic “fried egg” appearance, and result in biochemically abnormal surfactant that is dysfunctional [8].

Here, we provide evidence for a familial clustering of ILDs associated with a novel homozygous ABCA3 gene mutation; affected family members exhibited a variety of interesting clinical, radiological, and cellular features.

## Materials and methods

### Subjects

All members of the family described here were enrolled in a study protocol focused on identifying the genetic cause of ILD that affected some members of this family, which is from Italian origin.

As control groups for the determination of gene variant frequency, 242 healthy individuals (lab staff and blood donors), 30 patients with pulmonary alveolar proteinosis (PAP) and 113 patients with idiopathic pulmonary fibrosis (IPF), all of Italian descent, were enrolled.

The diagnosis of PAP was established by the presence of typical “crazy paving” pattern on chest high-resolution computed tomography (HRCT) and consistent morphology confirmed with a lung biopsy, or bronchoalveolar lavage fluid (BALF). Autoimmune PAP was defined on the basis of a positive test result for serum auto antibodies anti GM-CSF (GMAb). GMAb levels were measured by ELISA on patients’ sera which were previously collected and stored frozen (-20°C), as previously reported [9].

For the IPF patients, enrolment criteria included those described in the international consensus documents (ATS/ERS 2002), appropriate clinical picture coupled with a surgical lung biopsy or HRCT findings consistent with UIP (usual interstitial pneumonia) were required. No IPF patient has a positive family history. Ethical implications and confidentiality were discussed with family members; all individuals gave written informed consent before entering the study, which was approved by the ethical committee of the institutions involved. The ethical committee approved the use of human tissue for this investigation.

### Pulmonary function testing

When indicated, subjects were tested for FEV1/FVC, FVC,VC, single-breath diffusing capacity of carbon monoxide (DLCO), and arterial blood gases at rest and exercise.

### Genetic analysis

Genomic DNA was extracted from EDTA whole blood or dried blood spots (DBS) with the DNA Mini Kit® (Qiagen). DNA from IPF patients was extracted from paraffin embedded tissue specimens with the NucleoSpin Tissue kit (Machery-Nagel) according to the manufacturer’s protocol. After identification of the ABCA3 Gly964Asp mutation in the proband, genomic DNA of the parents and siblings was examined if available. ABCA3 Gly964Asp SNP detection was performed with the Taqman Assay on a Light cycler 480 (Roche), with the following primers and probe: forward primer: AGGTCCCGGGAACTGAGAA, reverse primer: CCATGCTGAGGCTGACCTT, reporter 1: VIC-CGAGTACGGCAGAACC, reporter 2: FAM-CGAGTACGACAGAACC, quencher: NFQ. In heterozygous and homozygous samples, each of the 30 coding exons of the ABCA3 gene including the donor and acceptor splice sites was amplified by PCR using exon specific primers [10], located in the flanking intronic regions and sequenced by direct sequencing using a CEQ8800 system (Beckman Coulter).

The five coding exons of SFTPC and the exon–intron boundaries were analyzed by direct DNA sequencing of polymerase chain reaction (PCR) products using specific primers as previously described [11].

### Bioinformatic analysis

Sequences were analysed using BioEdit software (7.0.5.3 version) and compared with the reference sequences NM_001089, for the ABCA3 gene, and NM_003018.3, for the SFTPC gene. Multiple sequence alignment was performed with the ClustalW software. For predictive analysis of mutation effects, the web-based tool FANS (Functional analysis of novel SNPs and mutations in human and mouse genomes) was used [12].

### Western blot analysis

Total protein content was determined with the BioRad Protein Assay Kit (BioRad, Richmond, CA, USA). Four BALF (bronchoalveolar lavage fluid) samples containing 5 μg total protein each were prepared to detect Pro-SP-B and Pro-SP-C under reducing and SP-B and SP-C under non-reducing conditions. The proteins were separated on NuPage 10% Bis-Tris gels using a Novex X-cell II Mini Cell system (Novex, San Diego, CA, USA) and then transferred onto nitrocellulose membranes by Western Blot in Nupage Blot modules (Novex, San Diego, CA, USA). Membranes with proteins separated under reducing conditions were first incubated with pro-SP-B-antibody (recombinant anti-human from rabbit, C-terminal, charge 1/24/00, from Guttentag, USA), SP-C-antibody (recombinant anti-human from rabbit, charge 22/96, from Byk-Gulden, Konstanz, Germany), SP-B-antibody (recombinant anti-human from rabbit, charge C 329, from Byk-Gulden, Konstanz, Germany) and pro-SP-C-antibody (recombinant anti-human from rabbit, N-terminal, from Beers, USA). As second antibody we used horseradish peroxidase (HRP) conjugated goat Ig-G anti-rabbit from DIANOVA, Hamburg, Germany. The membranes were activated with the enhanced chemiluminescence assay before exposing them to x-ray films (Hyperfilm ECL, Amersham Biosciences, Buckinghamshire, UK). After development, films were scanned with the FluorSMulti-Imager and bands were analyzed using the software-program "Quantity One". In addition to the qualitative evaluation, a quantitative analysis was performed by multiplying the optical density with the average diameter of each band. Comparison with standard curves allowed approximate determinations of the amount of SP-B and SP-C in each patient sample [13]. HA-Tag, EEA1, calnexin, BiP and β-Actin in non-transfected and transfected A549 cells were detected by western immunoblot as previously described [14]. The following antibodies were used: primary antibodies, rat anti-HA-tag (Roche), rabbit anti-EEA1 (Acris Antibodies), rabbit anti-BiP (Cell Signaling), goat anti-calnexin (Santa Cruz); secondary antibodies, goat anti-mouse, goat anti-rabbit and rabbit anti-goat IgG antibodies (Dianova), conjugated to HRP; anti-β-Actin antibody (Santa Cruz), conjugated to HRP.

### Histochemistry

Hematoxylin and eosin-stained slides of lung surgical biopsies from patients A and B were available. Histopathological specimens were examined by pulmonary pathologists from other institutions and reviewed by the pulmonary pathologist at our center.

### ABCA3 Gene expression analysis

Total RNA was isolated from blood collected in PAX Gene RNA Tubes (PreAnalytix). Messenger RNA was reversed transcribed into complementary DNA with ThermoScript RT-PCR (Invitrogen) and used to measure messenger RNA expression levels relative to house-keeping β-actin mRNA (Human ACTB Gene Assay, Roche). Real Time PCR analysis was performed on a LightCycler 480 (Roche, Mannheim, Germany), by using two independent assays (assay 27 and assay 62). The primers for the real-time PCR assays were: 27 F (ccttcgtggacctgacctt), 27R (gctttctccagaataccgaaaa), used in combination with UPL (Universal Probe Library) #27 (Roche), and 62 F (gccagttccccagtagtcct), 62R (ttgctcagctccacactcat) used in combination with UPL #62 (Roche). The primer set 62 F/R was localized at the beginning (nucleotides 332 to 403, NM_001089.2 sequence) and 27 F/R at the end of ABCA3 mRNA (nucleotides 5598 to 5718, NM_001089.2 sequence). All PCR experiments were conducted in triplicate with reactions taking place in a final 10 μl volume, by adding the following: 0.2 μl forward primer (200 nM), 0.2 μl reverse primer (200 nM), 0.2 μl UPL probe, 5 μl LightCycler 480® Probe Master, 0.2 μl reference primer (Human ACTB Gene Assay, Roche), 0.2 μl reference probe (Human ACTB Gene Assay, Roche), 1 μl water, 3 μl cDNA diluted 1:50.

The “Advanced Relative Quantification” analysis provided by the LightCycler 480®software was used to generate reliable results, in terms of concentration ratio target/reference.

Total RNA was isolated from transfected cells using the High Pure RNA Isolation Kit (Roche). 1 μg of total RNA was reverse transcribed into cDNA using the QuantiTect Reverse Transcription Kit (Qiagen). Quantitative real time PCR was performed with the iQ™SYBR® Green Supermix (Bio-Rad) and the following primers: ABCA3-for 5’-*CGGGAAGACCACGACTTT*-3’, ABCA3-rev 5’-*GCTGCCGCACCTTTC*-3’, HPRT-for 5’*-CATTGTAGCCCTCTGTGTGC-*3’ and HPRT-rev5’-*CTGACCAAGGAAAGCAAAGTCTG*-3’ in an I-cycler real time PCR machine (Bio-Rad). Relative changes in ABCA3 mRNA expression were calculated using the 2^-ΔΔCT^ method after normalization to the housekeeping gene hypoxanthine phosphoribosyltransferase (HPRT).

### Plasmids

pUB6-ABCA3 vectors were constructed as previously described [15]. The ABCA3-G964D point mutation was introduced into the pUB6-ABCA3-WT vector using the Quick Change Site-directed mutagenesis kit (Stratagene) with the following primers: G964D-for 5’-*GCGAGTACGACAGAACCGTCGTG*-3’ and G964D-rev 5’-*CACGACGGTTCTGTCGTACTCGC*-3’.

### Cell culture

The human A549 epithelial cell line was obtained from DMSZ (German Collection of Microorganisms and Cell Cultures, Braunschweig, Germany). Cells were maintained in RPMI medium supplemented with 10% FBS. For all experiments, cells were seeded into 12-well plates and transfected with pUB6 vectors at 70% confluence with ExGen 500 (Fermentas). In mock-reactions, transfection reagent without plasmids was used. Cells were harvested 48 h after transfection.

Human HEK cell line was obtained from DMSZ. Cells were maintained in RPMI medium supplemented with 10% FBS. For stable transfection, cells were seeded into 12-well plates and transfected with pEYFP-*hABCA3* vectors at 70% confluence with ExGen 500. 24 h after transfection, selection was started by addition of 500 μg/ml G418. After reaching confluence, cells were singled out from the wells of a 96-well plate. Stable clones were selected by YFP fluorescence and western blot signal. For experiments, stable cells were seeded onto 12-well plates with or without coverslips and cultured for 72 h, at 37°C, 5% CO_2_.

### Immunofluorescence staining

Cells grown and transfected on 15×15 mm cover slips were fixed with 4% PFA, permeabilized with Triton-X 100 and stained with rat anti-HA-Tag (Roche), mouse anti-CD63 (LAMP3) (Chemicon) or goat anti-calnexin (Santa Cruz) primary antibodies. Alexa Fluor® 488 donkey anti-rat IgG (H + L), Alexa Fluor® 555 donkey anti-mouse IgG (H + L) and Alexa Fluor® 555 F(ab')_2_ fragment of goat anti-mouse or anti-goat IgG (H + L) secondary antibodies (Invitrogen) were used to visualize the signal. Coverslips were viewed with a Zeiss Axiovert 135 microscope and images were recorded with a Zeiss AxioCam MR camera and the Axiovision 3.1 software.

### Electron microscopy

Cells were fixed in 150 mM HEPES, pH 7.35, containing 1.5% formaldehyde and 1.5% glutaraldehyde at room temperature for 30 min and then at 4°C over night. After dehydration in acetone cells were embedded in EPON. 50 nm sections were stained with 4% uranyl acetate and lead citrate [16] and observed in a Morgagni TEM (FEI). Images were taken with a 2 K side mounted Veleta CCD camera.

### Lipid analysis of cultured cells

Cells were extracted in the presence of not naturally occurring lipid species as internal standards and crude lipid extracts were quantified by direct flow injection electrospray ionization tandem mass spectrometry (ESI-MS/MS) in positive ion mode [17,18]. Lipid species were annotated according to the shorthand notation of lipid structures [19]. Results are presented as total phospholipids or free cholesterol, expressed as nmol/ml.

### Statistics

Comparisons of multiple groups were made using one-way repeated measure ANOVA with Tukey’s post hoc test, comparisons of two groups were made using the students t-test. Results are presented as mean + S.E.M. from a minimum of three different experiments. All tests were performed using GraphPad Prism 4.0 (GraphPad Software). P-values of less than 0.05 were considered statistically significant.

## Results

### Patient A (the proband)

**Figure 1:**
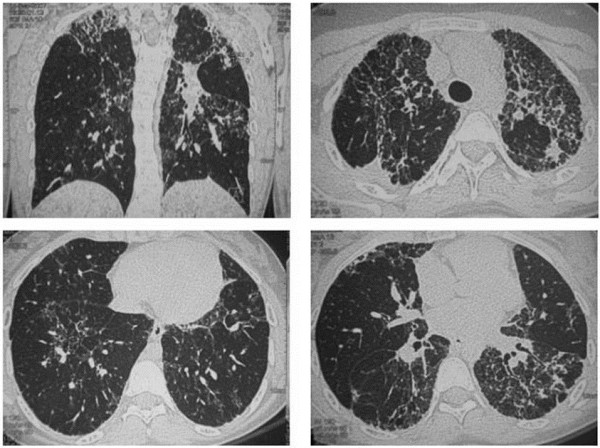
**Patient A - chest CT at diagnosis.** High-resolution CT scan demonstrates fibrotic architectural distortion with intra and inter-lobular thickening, associated with apical honey-combing. No ground glass opacity is present.

**Figure 2:**
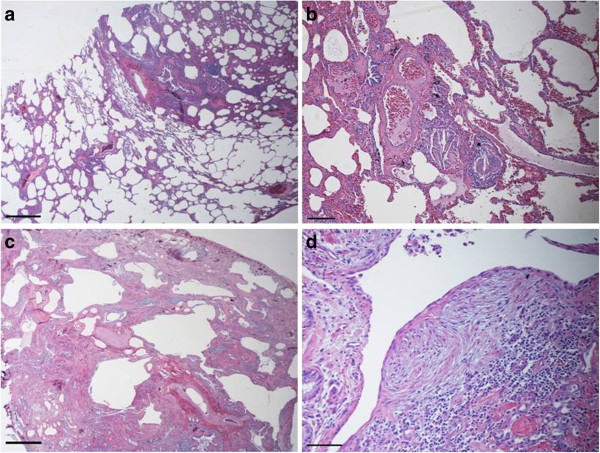
**Patient A Hematoxylin and Eosin staining.** Two surgical samples were taken from the upper and the lower left lobes. The sample from the upper lobe **(a, b)** shows limited architectural changes, with centrilobular inflammation and fibrosis. Fibroblastic foci and small aggregates of multinucleated giant cells engulfing cholesterol clefts are visible in centrilobular areas. Granular pink material is visible in some alveolar spaces **(b)**. The sample from the lower lobe **(c, d)** shows diffuse microcysts, fibrosis and smooth muscle proliferation, without residual normal tissue. Fibroblastic foci and bronchiolar metaplasia are prominent **(d)**. **(a)** magnification 2x, scale bar 0,5 cm; **(b)** magnification 10x, scale bar 200 micron; **(c)** magnification 2x, scale bar 0,5 cm; **(d)** magnification 20x, scale bar 30 micron.

**Figure 3:**
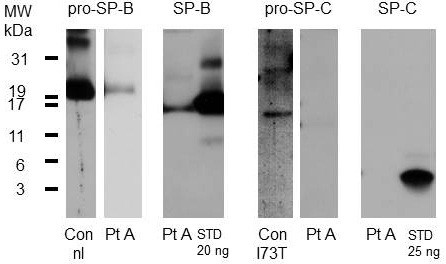
**Analysis of pro and mature SP-B and SP-C in BALF.** Patient A (Pt A). Molecular weights (MW, kDa) are indicated on the left side. 5 μg of total protein was added to each lane from lavage of a patient. All bands were analyzed under non-reducing conditions. A small amount of pro-SP-B was detected at 21 kDa in Pt A (second lane from left). As positive control pro-SP-B of a lavage from a healthy subject (Con, nl) is given (first lane from the left). SP-B was detected in fair amounts (third lane compared to the standard (STD) of which 20 ng was applied). Aberrant proforms of SP-C were not detected (third lane from the right). As positive control pro-SP-C of a lavage from a subject with SFTPC mutation I73T, which usually have aberrant pro-SP-C at about 14 kDa (Con, I73T) is given (fourth lane from the right). No SP-C was detected (second lane from the right; compared to the standard STD of 25 ng applied to the first lane from the right).

A 16 year-old, girl who never smoked was referred to the IRCCS Policlinico S. Matteo hospital for ILD causing a progressive exertional dyspnoea associated with digital clubbing. The patient medical history indicated that by the age of 2 years, pulmonary aspiration was suspected as a consequence of gastroesophageal reflux and sliding hiatal hernia; this was confirmed by pH-metry, contrast radiography analysis and gastroscopic examination. By the age of 4, her HRCT showed bronchi with thick walls and small bronchiectasis. One year later, ventilation*-*perfusion lung scintigraphy revealed a perfusion defect involving both upper lobes. By the age of 13, a chest HRCT scan revealed diffuse ILD characterized by a fine reticular pattern alternating with areas of normal lung tissue and some areas with irregularly distributed small sized bullae. BAL revealed numerous lipid*-*laden macrophages, whereas lung function testing showed a restrictive syndrome (Table 1) with a marked reduction in DLCO. A diagnosis of PAP was suspected and the patient was referred to our center. The chest HRCT showed fibrotic ILD, with an atypical pattern, characterized by features of IPF/UIP, fibrotic NSIP, and centrilobular fibrosis (Figure 1). The ILD was predominant in the upper lobes, with relative sparing of the lower lobes. The serum GMAb test was negative and the serum LDH level was within the normal range. A VATS (Video-Assisted Thoracic Surgery) lung biopsy revealed pulmonary fibrosis with a UIP pattern (Figure 2). To investigate whether the condition was associated with surfactant abnormalities, hydrophobic surfactant proteins were analyzed by Western blotting of BALF. A small amount of pro-SP-B was detected, whereas SP-B was detected in fair amounts. No aberrant proforms of SP-C were detected, but importantly, no SP-C was detected (Figure 3)*. *Clinical course: the patient is currently in follow up, and in stable conditions. Cycles of low dose prednisone and macrolides at anti-inflammatory doses have been administered.

**Table 1:** Pulmonary function test of the ABCA3 AA patients and GA and GG carriers

	**Homozygous AA**			**Heterozygous GA**	**Homozygous GG**	**P**
	**Patient A**	**Patient B**	**Patient C**	**(n = 10)**	**(n = 6)**	
				**mean ± sd**	**mean ± sd**	
**FVC (L)**	2.01	2.60	4.09	2.91 ± 0.83	3.85 ± 0.66	0.0338
**FVC (%)**	52	68	88	95.86 ± 6.95	109.55 ± 24.68	0.1148
**FEV1 (L/s)**	1.82	1.95	3.18	2.34 ± 0.68	3.07 ± 0.62	0.0500
**FEV1 (%)**	54	65	86	97.19 ± 12.42	102.95 ± 19.02	0.4726
**FEV1/VC**	90.55	73.03	77.75	71.30 ± 6.66	70.45 ± 5.87	0.8004
**FEV1/VC (%)**	107	95	100	92.83 ± 11.47	87.55 ± 6.29	0.3209

The family members carrying one A allele had a mean FVC absolute value slightly reduced compared to WT family members (p = 0.0338), but no difference when values were expressed as % predicted.

### Genetic analysis

**Figure 4:**
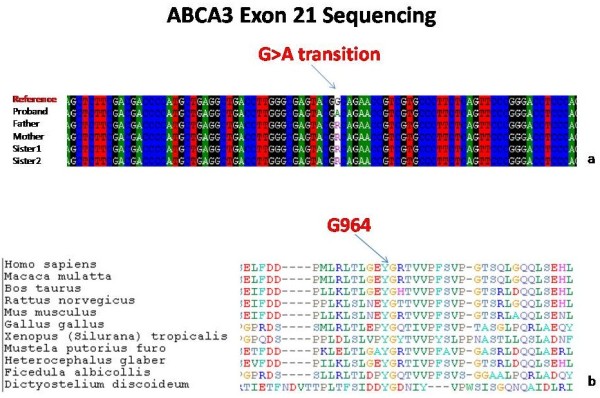
**ABCA3 gene sequence analysis. a)** Sequence analysis of ABCA3 G > A transition at nucleotide 2891 in Patient A, her parents and sisters. **b)** Partial amino acid alignment of ABCA3 sequences (codons 949–986), showing that Gly964 (indicated by an arrow) is extremely conserved.

Sequence analysis of all 30 coding exons of the ABCA3 gene revealed the presence of a novel homozygous G > A transition at nucleotide 2891, localized within exon 21, resulting in a glycine to aspartic acid change at codon 964 (Figure 4). Bioinformatic analysis of this mutation identified a high risk associated with the A allele, which putatively causes the abolishment of a protein domain.

**Figure 5:**
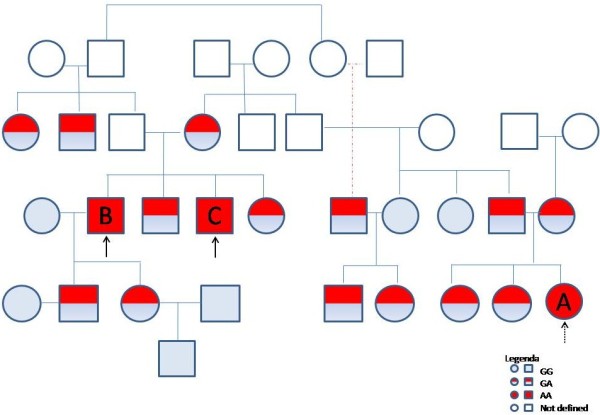
**Family tree.** The family genetic screening by the Taqman assay resulted in the identification of two other paternal members (black arrows), who were homozygous for the same ABCA3 Gly964Asp mutation. The proband is indicated by the dashed arrow.

A search for this ABCA3 mutation resulted negative in 195 Italian healthy individuals, in 30 Italian patients with autoimmune pulmonary alveolar proteinosis, and 113 patients with idiopathic pulmonary fibrosis (data not shown). Both parents and two siblings of the proband were heterozygous for this ABCA3G/A mutation (Figure 5). They had no symptoms or signs of ILD. Interestingly, the parents disclosed a history of consanguinity, being second*-*degree blood relatives. On that basis, we extended the genetic analysis to another 18 members of the family. Figure 4 shows the pedigree of the family: we identified 10 ABCA3 G/A heterozygous carriers and 6 ABCA3 G/G homozygous carriers. Medical history and analysis of these 16 family members did not reveal any clinical abnormalities; all had normal pulmonary function test results (Table 1) and chest X-rays. Interestingly two subjects (herein after referred as patients B and C) were homozygous for the ABCA3 Gly964Asp mutation.

Sequence analysis of SFTPC coding exons detected two common non-synonymous polymorphisms: rs4715 heterozygous C/A and rs1124 homozygous AA, inside exon 4 and exon 5 respectively (Table 2). In patient A, B and C, no mutation was detected in SFTPA and SFTPB gene sequence; moreover the long PCR of SFTPC gene did not reveal any large deletion (data not shown).

**Table 2:** SFTPC and ABCA3 genetic analysis

	SFTPC gene*	SFTPC gene*	**ABCA3**
	**c.413C > A (rs4715)**	**c.557 G > A (rs1124)**	**2891 G > A**
	**Exon 4**	**Exon 5**	**Exon 21**
Proband	CA	AA	AA
Mother	CC	GA	GA
Father	CA	GA	GA
Sister 1	CC	GA	GA
Sister 2	CA	GA	GA
Patient B	CC	GA	AA
Patient C	CC	GA	AA

*Common non-synonymous SFTPC polymorphisms:C/A at rs4715 yields Thr138Asn; G/A at rs1124 yields Ser186Asn.

In all tested subjects, including patients B and C, the serum GMAb test was negative.

### Patient B

Patient B was a 57 year old male, non-smoker. In 2005, at the age of 52, he started to complain of cough and dyspnea on exertion. Chest X-ray showed bilateral areas of interstitial thickening. After several cycles of antibiotic therapy which proved ineffective, a HRCT scan of the thorax was performed, revealing a bilateral patchy interstitial fibrosis associated with diffuse ground glass opacities, the latter prevailing on the fibrotic component. Minimal apical honeycombing was also present.

**Figure 6:**
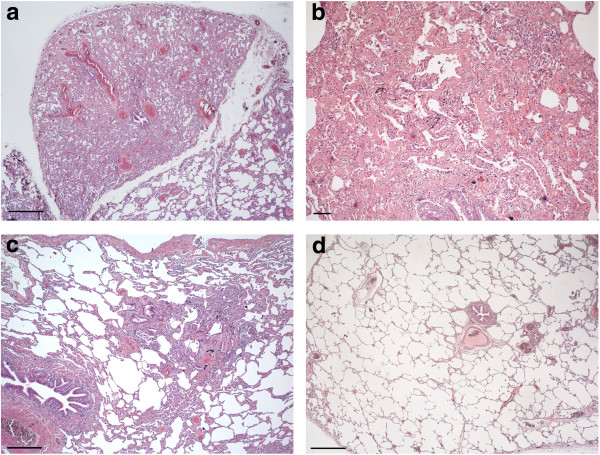
**Patient B Hematoxylin and Eosin staining.** A single sample of lung tissue was obtained. The site of the biopsy could not be documented. **a)** Low power evaluation showed a patchy fibrotic process, characterized by areas of interstitial scarring (upper left) in an otherwise uninvolved alveolar background; **b)** fibrotic area characterized by an NSIP pattern; **c)** isolated centrilobular fibrosis and inflammation; **d)** areas with normal lung parenchyma.

The pulmonary function test showed a mild restrictive syndrome with marked reduction in DLCO (49% predicted). Gas exchange at rest was normal. The 6MWT (six Minutes Walking Test) showed reduced tolerance to physical exertion in hemoglobin oxygen desaturation. Collagen-vascular diffuse lung disease was excluded and in February 2006 the patient underwent a VATS lung biopsy, revealing interstitial pulmonary fibrosis, suggestive of collagen vascular disease or hypersensitivity pneumonia, characterized by areas of interstitial scarring in an otherwise uninvolved alveolar background. The patient was then referred to our center, and lung specimens were reviewed. In most lobules, fibrosis was limited to the centrilobular areas; however complete scarring of some lobules was observed in the subpleural region. In the centrilobular areas, lesions of variable severity were observed, spanning from simple lymphocytic infiltration of the submucosa of terminal bronchioli, to inflammatory infiltrates associated with mild fibrosis, limited to the bronchiolar walls, to severe bronchiolar scarring and fibrosis of the alveolar septa surrounding the small airways associated with peribronchiolar metaplasia and mucus plugging. The fibrosis was dense, eosinophilic and homogeneous. Portions of the lobules not involved in the scarring were unremarkable. Membranous bronchi showed lymphocytic infiltrates and mild submucosal scarring. Sparse interstitial lymphocytic infiltrates were present (Figure 6). When consanguinity with patient A was disclosed, the ABCA3 gene was sequenced and homozygosity for the Gly964Asp mutation was identified. Western blot analysis of BAL fluid detected no pro-SP-B and low SP-B levels (53 ng/ml). No SP-C was detected.

**Figure 7:**
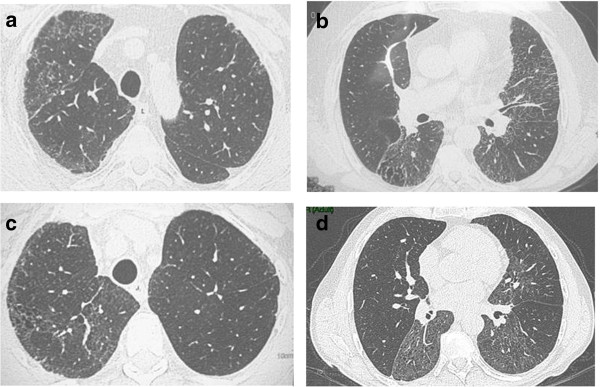
**Thorax HRTC Scan of the two brothers with the homozygous ABCA3 mutation. a**, **b)** Patient B. Diffuse areas of reticular ground glass opacities, prevalent in the upper right lobe. Initial honeycombing in the dorsal segment. Diffuse pleural thickening. **c** and **d)** Patient C. The involvement of the upper lobes is very similar to that of the brother, patient B.

The patient has been in follow up at our center for the last five years, treated with low dose prednisone and low dose azitromycin, with minimal progression of lung function impairment. The last HRTC scan showed the disappearance of ground-glass areas with persistence of fibrosis (Figure 7a,b).

### Patient C

Patient C, brother of patient B, was a 52-year old, mild former smoker, who did not complain of respiratory symptoms and, interestingly, his ILD was diagnosed following the genetic analysis.

His clinical history was remarkable for acute myocardial infarction, treated with coronary revascularization three years before. The chest X-ray taken at that time was considered within normal limits. Pulmonary function testing documented normal lung volumes (Table 1), but with a marked reduction in DLCO (56% of predicted). The 6MWT showed reduced exercise tolerance with oxyhemoglobin desaturation.

We did not perform any invasive diagnostic procedures, because of the patients’ chronic ischemic cardiac disease. His HRTC chest scan revealed a pattern very similar to that found in his brother, with areas of reticular ground glass opacities and initial honeycombing (Figure 7c,d). Parenchymal abnormalities had a bilateral and asymmetrical distribution, with a clear separation between preserved and altered parenchyma. Patient is currently in follow up, and in stable conditions. No specific treatment has been so far administered.

### Gene expression analysis

To determine the effect of the ABCA3 G > A transition at nucleotide 2891 on gene transcription, we performed a Real Time PCR relative quantification analysis by using two independent assays. We analysed ABCA3 mRNA from three healthy subjects with ABCA3 GG genotype, four GA heterozygous subjects and the two ABCA3 AA homozygous brothers. No differences in mRNA levels between heterozygous, non-mutated and mutated homozygous subjects were detected in peripheral blood (mean concentration ratios target/reference were 0.42, 0.50, 0.46, respectively with assay #62 and 0.16, 0.17,0.20, respectively with assay #27).

### Expression, intracellular processing, and cellular effects resulting from ABCA3 G > A transition at nucleotide 2891

**Figure 8:**
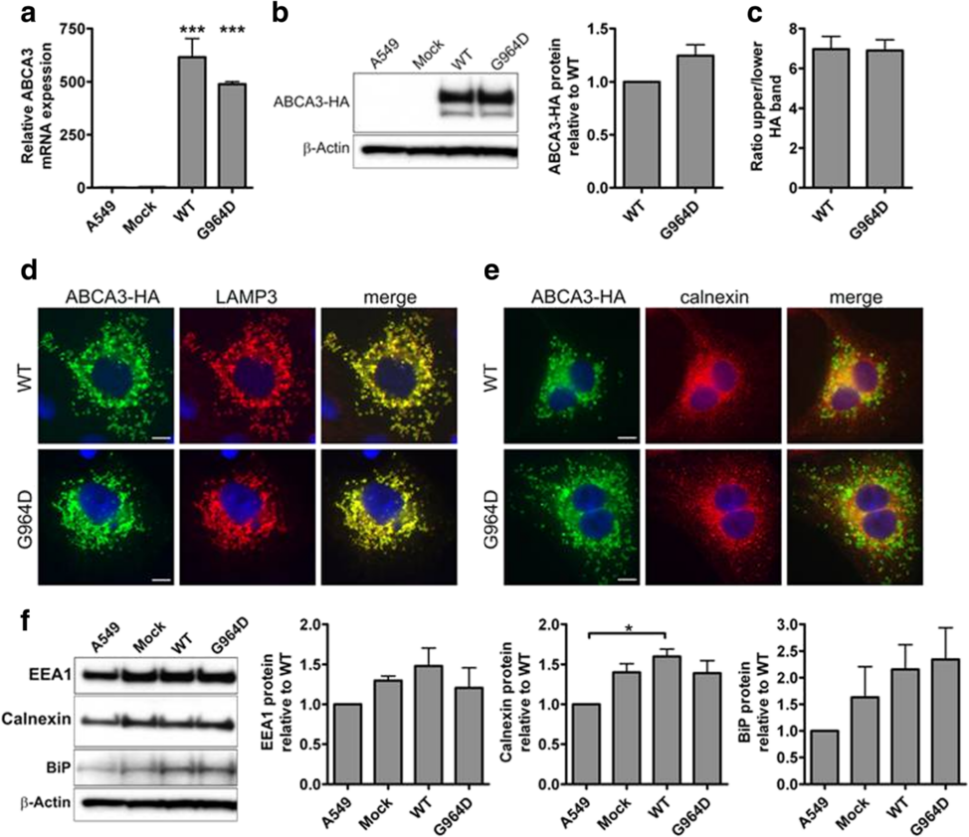
**Cellular effects of 48 h transient ABCA3 expression in A549 cells. a.** ABCA3 mRNA expression levels analyzed by quantitative real time PCR (*P* < 0.001 for WT/G964D vs. A549/Mock). **b.** Western immunoblot analysis of HA-tagged ABCA3 in total cell lysates. β-actin was used as a loading control. **c.** Ratio of the upper/lower ABCA3 processing form. **d.** Co-immunostaining of HA-tagged ABCA3 with the lysosomal (lamellar body) marker LAMP3. **e.** Co-staining of HA-tagged ABCA3 with the ER marker calnexin. **f.** ER (calnexin, BiP) and early endosome markers (EEA1) in cells transiently transfected with ABCA3. β-actin was used as a loading control. Scale bars 7.5 μm, *P < 0.05.

Transient transfection of A549 cells with DNA coding for ABCA3-WT and ABCA3-G964D induced ABCA3-mRNA transcription 500 to 600-fold compared to non-transfected cells (Figure 8a). Protein concentrations of HA-tagged ABCA3 in ABCA3-WT and ABCA3-G964D transiently transfected A549 cells were similar (Figure 8b). Intracellular processing as assessed by western immunoblotting did not differ between ABCA3-WT and p.G964D, both proteins were found to have a 150 kDa as well as a larger 190 kDa processing form (Figure 8b). The ratio of upper to lower bands was not significantly different in ABCA3-WT and p.G964D cells (Figure 8c). Immunofluorescence staining of transfected A549 cells revealed a mostly vesicular ABCA3 pattern, which colocalized with the lamellar-body marker LAMP3 for both HA-tagged ABCA3-WT and G964D proteins (Figure 8d). This is in concordance with the native localization of ABCA3 in the outer membrane of lamellar bodies in alveolar type II cells. Almost no colocalization was observed for ABCA3-WT and p.G964D with the ER-resident chaperone calnexin, so ER-retention due to protein-misfolding can be excluded (Figure 8e). Expression of the p.G964D mutation did not induce the early-endosomal marker (EEA1), the ER chaperone calnexin or the ER-resident stress marker BiPwhen compared with the ABCA3-WT protein (Figure 8f).

Similar results for the intra-cellular processing and the lack of induction of ER stress were obtained with stably transfected HEK cells with DNA coding for ABCA3-WT and ABCA3-G964D (Additional file 1: Figure S1).

The G964D mutation decreases ABCA3-induced intracellular accumulation of phospholipids and free cholesterol in HEK cells.

**Figure 9:**
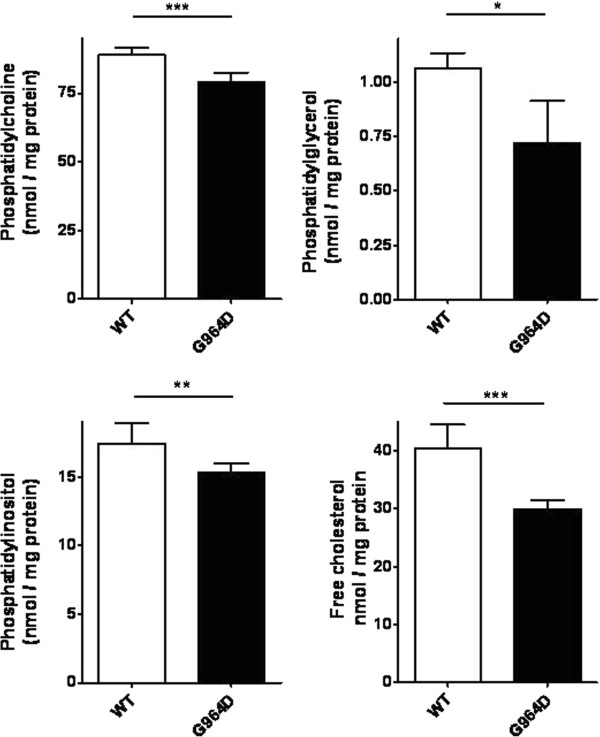
**Levels of phospholipids and free cholesterol in HEK cells.** Shown are cellular contents of phosphatidylcholine, phosphatidylglycerol, phosphatidylinositol, and free cholesterol in HEK cells expressing wild type ABCA3 and ABCA3 G964D, respectively. Values are given in nmol/mg protein. *P < 0.05, **P < 0.01, ***P < 0.001.

Phospholipids and cholesterol were determined in HEK cells stably transfected with vectors coding for ABCA3-WT and G964D. Phosphatidylcholine (PC) was the most abundant phospholipid (approximately 50% of total phospholipids), while phosphatidylinositol (PI), phosphatidylserine, phosphatidylethanolamine, and sphingomyelin were present in smaller amounts. Cellular content of PC, phosphatidylglycerol (PG) and PI were significantly increased in cells expressing wild type ABCA3 when compared to G964D stably transfected HEK cells (Figure 9).

**Figure 10:**
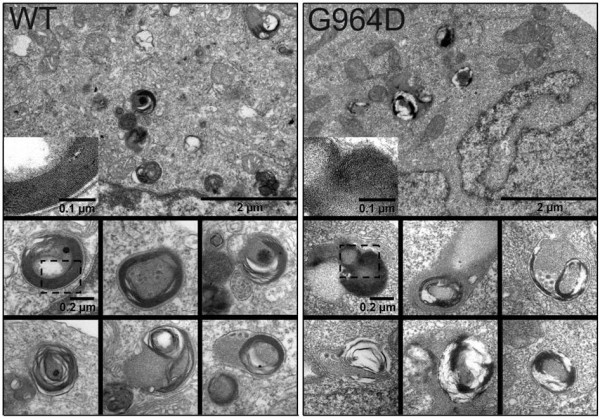
**Transmission electron microscopy of stable transfected HEK293-cells.** Top: overview of one cell showing cytoplasm, containing mitochondria (light grey) and organelles resembling lamellar bodies (dark grey). Bottom: single ‘lamellar bodies resembling organelles’ in higher magnification. The boxed area is shown in the top image as inset in higher magnification. Note the concentric arrangement of the lamellae in the ABCA3-WT strain. Dark appearing lamellae are mostly wound around a centre of less electron density and appear as parallel lines in the high magnification. In the strain G964D these organelles appear less organized: lamellae are often not arranged perfectly parallel (see inset), and the concentric organisation is not as distinct as in the WT.

Electron microscopy of both stably transfected WT and G964D HEK cells possessed organelles which resemble lamellar bodies (Figure 10). Whereas these organelles appeared in different states of organisation, G964D transfected HEK had less mature and more variable appearing organisation of the organelles than the WT.

Taken together, these results suggest that transport function of ABCA3 with the G964D mutation is severely impaired especially with regard to the sorting of important surfactant phospholipids and FC, as well as quality of lamellar body formation.

## Discussion

An increasing body of evidence suggests that inherited or acquired disorders associated with the surfactant system may result in the development of ILD, either acute or chronic. Among the different genes involved in these processes, the ABCA3 gene encodes a 1704 amino acid protein of the ATP binding cassette transporter family. It is mainly expressed in the lungs at the limiting membrane of the lamellar bodies of alveolar type II cells; loss of normal ABCA3 function is associated with lung disease [20,21].

There is a wide spectrum of mutations in the ABCA3 gene, with more than 150 described so far. Some mutations correlate with forms of severe respiratory distress in newborns and death within the first months of life, whereas other mutations are associated with milder clinical forms, such as ILD, desquamative interstitial pneumonitis or NSIP presenting in childhood [1]. A compound heterozygous mutation involving a substitution of valine for glutamic acid in codon 292 (E292V) on at least one allele has been identified in children with chronic ILD. These children survived well beyond infancy and in one case to early adulthood. The onset of symptoms in the majority of these patients was at birth, in the neonatal period, or in infancy (<1 year), with histopathologic diagnoses of PAP or DIP [5].

Compound heterozygous mutations in ABCA3 have also been identified in adults with chronic ILD, as well as in one adolescent patient who presented at age 15 with a 6-month history of exercise intolerance, chest discomfort, and histologic features of UIP [22]. ABCA3 may also act as a modifier gene for the phenotype associated with an SFTPC mutation, as heterozygosity for ABCA3 mutations in severely affected infants with SFTPCI73T leads to more severe lung disease than family members with only the I73T mutation [23]. Recently a full-term baby girl, who developed severe chronic lung disease and eventually became ventilator-dependent via tracheotomy, was reported [24]. Sequence analysis revealed that the patient was a homozygous carrier of the loss-of-function mutation p.Trp308Arg (c.922 T > C) at exon 9 of the ABCA3 gene, without any additional abnormalities in SFTPB, SFTPC and CSF2RA genes.

The ABCA3 G964D mutation identified in our patients has never been described previously and it shows an autosomal recessive pattern of inheritance, as suggested by evidence that lung disease develops only in homozygous carriers. The ABCA3 G964D mutation was not detected in a large control group of healthy subjects, thus it is unlikely that it is a polymorphism, but rather a rare variant. Recently, Flamein et al. [25] reported a case where an infant died from neonatal respiratory distress, which carried a ABCA3 G964S mutation in compound heterozygosity with a L462R mutation. Together with the fact that aminoacid G at position 964 is conserved in many species, this suggests that this position is critical for correct function of the protein. When we performed the pedigree analysis we took into account the high degree of inbreeding that characterized this family. From this point of view, consanguinity was implicated in the susceptibility to familial ILD, with cousin marriages sharing the same predisposing allele inherited from a common ancestor. Patients with a hereditary ABCA3 deficiency do not share a typical histopathological pattern. It has been suggested that histology patterns of diffuse lung disease in pediatric cases differ from those of adults. The reported fibrotic evolution of ABCA3 pediatric lung disease [26] could in part support the hypothesis that the different pattern observed in our patients correspond to different phases of the disease evolution, as suggested by the presence of similar histological features in less affected areas in patients A and B. On the other hand, the evidence of a more advanced disease stage in the younger patient (patient A) contradicts this hypothesis.

To the best of our knowledge, only one other case of an adolescent patient with an UIP pattern of pulmonary fibrosis associated with an ABCA3 mutation has been described [22]. Our patient A exhibited a similar distribution of pulmonary lesions with prevalence in the upper lobes and relative sparing of the lower lobes. These features are actually in contrast with the HRCT scan pattern of common UIP/IPF, which is typically characterized by predominant lower lobe involvement, with peripheral/subpleural honeycombing and reticular opacities.

In marked contrast, the histology pattern observed in patient B was characterized by a patchy fibrotic process, with NSIP-like lobular scarring, while the HRTC scan of both adult patients (B and C) shared a very similar pattern, with areas of reticular ground glass opacities and initial honeycombing. They were both diagnosed at a much older age, and the morphology and radiology features are consistent with a milder form of disease.

In an attempt to further elucidate the consequences of the ABCA3 mutation on surfactant metabolism and in turn the development of ILD, we designed a series of experiments based on a cell culture model system for alveolar epithelial cells. Transient cellular expression of wild type and mutated G964D ABCA3 in alveolar epithelial cells resulted in enhanced expression of normal and mutated protein which did not differ in size and molecular form, intracellular processing or localization into lamellar body like structures. No differential retention in the endoplasmic reticulum (ER) was observed and the extent of expression of ER or early endosome stress markers was not different, suggesting no abnormalities were induced by the mutation in the folding and assembly of these proteins in the ER or their sorting to late endosomes and lysosomes. Similar results on ABCA3 expression, intracellular processing and the lack of induction of ER-stress were obtained for stably transfected HEK-293 T cells.

The substrates believed to be transported by ABCA3, i.e. phosphatidylcholine and cholesterol, were substantially enriched in HEK cells expressing wild type ABCA3 in comparison to non-transfected cells, demonstrating induction of ABCA3 specific activity with transfection of the transporter into the cells (data not shown). This result resembles findings from A549 cells expressing GFP-tagged ABCA3 [27]. We further demonstrated reduced level of the lipids characteristic for pulmonary surfactant, i.e. phosphatidylcholine, phosphatidylglycerol, phosphatidylinositol and free cholesterol, in those cells that carried the G964D – ABCA3. In accordance with these results it was also shown previously that phosphatidylcholine accumulation in cells expressing ABCA3 with the walker A motif mutant N568D was diminished [27]. However, in A549 cells, no increased free cholesterol levels have been observed. Accumulation of free cholesterol due to expression of ABCA3 in HEK cells is in line with the free cholesterol transport activity that has been reported for ABCA3 [28].

By electron microscopy lamellar bodies, as characteristic in type II alveolar epithelial cells in the lungs, were clearly induced and visualized in both stably transfected WT and G964D HEK cells. However G964D cells appeared to have a more variable and less mature organisation of these organelles than the WT. This finding is concordant with the functional transport defect for phosphatidylcholine, phosphatidylglycerol, phosphatidylinositol and free cholesterol observed in the cells carrying the G964D – ABCA3 mutations and described above.

Therefore, based on these cell culture data, disordered lipid composition, reduced and faulty lamellar body organization due to the G964D – ABCA3 mutation may represent mechanisms involved in the pathogenesis on ILD in this family, whereas misfolding, aberrant processing, mislocalization and ER-stress can be excluded as factors contributing to the development of ILD by the G964D mutation. However, as discussed below, ER stress seems not to be mandatory for an epithelial-mesenchymal transition (EMT) in ABCA3 mutation- associated ILD: accumulation of lipid species, as happening in ABCA3 impairment, might *per se* induce EMT [29].

Onset and progression of ABCA3-related ILDs might be influenced by external stress factors such as respiratory infections. Several respiratory viruses, as well as other viruses, especially Epstein-Barr Virus and Herpesvirus-8, have been implicated in ILD exacerbation [30]. Recently, Kaltenborn and coworkers [29] demonstrated that respiratory syncytial virus (RSV), one of the most common respiratory pathogens in infants, children and adults, could substantially drive a pulmonary epithelial cell phenotype into a fibroblast like phenotype, particularly in cells harboring ABCA3 mutations and infected by RSV. In those cells they observed alterations in cell morphology, loss of epithelial cell properties and function, and gain of mesenchymal, fibroblast-like characteristics suggesting that a genetic predisposition of epithelial cells, due to ABCA3 mutations, creates a permissive environment for the development of EMT, a process believed to play a central role in the development of pulmonary fibrosis.

As it is known that ILD induced by SFTPC mutations in children [5] and in adults [31] may be aggravated by ABCA3 mutations present in a heterozygous state, we hypothesized that alterations in SFTPC might have contributed to disease severity. We found two common non-synonymous SFTPC polymorphisms rs4715 (C/A Thr138Asn) and rs1124 (G/A Ser186Asn) in patient A, which are in strong linkage disequilibrium [32]. Thus we cannot exclude that the different presentation of the disease in patient A in comparison with patients B and C was due to the presence of these SFTPC variations (Table 2).

The BALF analysis of our patients demonstrates the lack of mature SP-C in alveolar spaces, which could be a further consequence of abnormalities in the lamellar bodies resulting from ABCA3 mutations. Mature SP-B and precursors of SP-C are delivered together with lipids by multivesicular bodies to lamellar bodies and subsequently secreted into the alveolar space after final proteolytic remodeling of the N-terminal SP-C propeptide [33]. This process may be impaired by ABCA3 defects. Bioinformatic analysis of the homozygous ABCA3 mutation suggested unaffected transcription levels of the gene, however with abolishment of a protein domain. In fact each aminoacid has its own specific size, charge, and hydrophobicity-value. The original wild-type residue and newly introduced mutant residue differ in these properties. The mutant residue is bigger than the wild-type residue. The wild-type residue was neutral, the mutant residue is negatively charged. The wild-type residue is a glycine, the most flexible of all residues. This flexibility might be necessary for the protein's function. Mutation of this glycine can abolish this function. It is possible that the residue is needed at this position to make a special backbone conformation or to facilitate movement of the protein. The mutation introduces a less flexible residue thereby disturbing this conformation or movement. Although little is known about the role of this domain for ABCA3 routing and function, the change of glycine to aspartic acid in the transporter could result in severely impaired phospholipid transport function which in turn could affect surfactant processing and contribute to the development of ILD in this family.

Familial interstitial pneumonia has emerged as a distinct clinical phenotype [34,35], characterized by an underlying pulmonary transcriptional profile markedly different from that of sporadic fibrosis [36]. The search for mutations in known candidate genes, including SFTPA2, SFTPC [37] and genes encoding for telomerase components (TERT, TERC) accounts for approximately 22% of these cases [38]. In contrast, in sporadic fibrosis, only 3–4% of cases are estimated to be caused by these genes. Moreover very recently the ATP11A gene, which encodes for another ATP-binding cassette transporter, has been identified as a risk factor for fibrotic idiopathic interstitial pneumonias in a genome-wide association study on 1616 cases [39]. In spite of the limitations of this paper, including the poor assessment of the family members (related to their domicile very far from Pavia), we believe that our results would demonstrate that ABCA3 mutations must be considered additional candidates that may cause familial fibrosis and thus we suggest that ABCA3 be included in genetic testing in cases of familial pulmonary fibrosis.

## Abbreviations

6MWT: Six minutes walking test; ABCA3: ATP binding cassette member A3; BALF: Bronchoalveolar lavage fluid; DLCO: Diffusing capacity of carbon monoxide; EMT: Epithelial-mesenchymal transition; ER: Endoplasmic reticulum; FEV1: Forced expiratory volume in the 1st second; FVC: Forced vital capacity; GMAb: Autoantibodies anti GM-CSF; GM-CSF: Granuloyte Monocyte-colony stimulating factor; HRCT: High-resolution computed tomography; ILD: Interstitial lung diseases; IPF: Idiopathic pulmonary fibrosis; LDH: Lactate dehydrogenase; NSIP: Non-specific interstitial pneumonia; PAP: Pulmonary alveolar proteinosis; PCR: Polymerase chain reaction; RSV: Respiratory syncytial virus; SFTPA: Gene for surfactant, pulmonary-associated protein A; SFTPB: Gene for surfactant, pulmonary-associated protein B; SFTPC: Gene for surfactant, pulmonary-associated protein C; SP: Surfactant-associated proteins; TERC: Telomerase RNA component; TERT: Telomerase reverse transcriptase; UIP: Usual Interstitial pneumonia; VATS: Video-assisted thoracic surgery; VC: Vital capacity.

## Competing interests

The authors declare that they have no competing interests.

## Authors’ contribution

IC performed the gene and gene expression analysis and drafted the manuscript, MZ performed the bioinformatic analysis, FM and ZK coordinated clinical diagnosis, PM performed the histochemistry analysis, RD performed the radiology investigation, EK and SF and RZ performed the Western Blot analysis and the cell transfection experiments, GL performed lipid analysis ofthe cells, JH and CW prepared electron micrographs and analyzed them. MG coordinated Western Blot lipidomic analysis and the cell transfection experiments and drafted and supervised the manuscript, ML drafted and critically revised the manuscript and supervised the research. All authors read and approved the final manuscript.

## Supplementary Material

Additional file 1 Supplementary methods. Click here for file
