# Supplementary material for: A large kindred of pulmonary fibrosis associated with a novel ABCA3 gene variant
Source: Respir Res. 2014 Apr 15;15(1):43. doi: 10.1186/1465-9921-15-43 (PMC4021316; doi:10.1186/1465-9921-15-43)
Supplement: Additional file 1 — Supplementary methods. [file 1465-9921-15-43-S1.docx]

**Supplementary methods:**

**SFTPC long PCR**

DNA was extracted from 200 μL of EDTA-blood with Qiamp blood minikit (Qiagen, Milano, Italy).

Long PCR assay was performed with a 50 μL reaction volume containing 5 μL of distilled water, 100 ng of DNA, 5 μL of 10x PCR Tuning Buffer with Mg^2+^ (2 mM), 2.5 μL of dNTPs (10 mM each), 400 nM of each primers (SFTPCfor 5’-*CCAGTGGGGACAGAGTTTCC*-3’ and SFTPCrev 5’-*TAGGGAAATGAGCTCGCTGG*-3’), and 2 U of PCR extender polymerase (5 U/μL, PCR Extender System) (5PRIME, Hilden Deutschland). The PCR product size was 3978 bp.

The amplification conditions for the PCR reaction were as follow 1cycle of 93°C for 3 minute, 10 cycles of 93°C 15 seconds, 62°C for 30 seconds and 68°C for 8 minutes, 8 cycles of 93°C 15 seconds, 62°C 30 seconds and 68°C 8 min + 20 seconds every cycles.

**SFTPA and SFTPB Sequencing.**

We sequenced all the 5 exons of SFTPA gene and all the 10 exons of SFTPB gene. Amplicons were directly sequenced in an automatic genetic analyzer (CEQ 8800 Genetic Analyzer; Beckman-Coulter). Primers used are shown in table S1 and S2.

| **SFTPA Exon** | **Primer** | **Product size** |
| --- | --- | --- |
| **1** | ACACTATGCCCATTTCCTGC | 219 |
|  | GCTGGTCCTCTCTGCCTG |  |
| **2-3** | TGACAGAGCACAGTGGGG | 759 |
|  | TGTAACTGACTTCAGGGTCGC |  |
| **4** | GCAGATGGCAAAACACCTG | 218 |
|  | AGAATGAGGGGAATTTGTGG |  |
| **5_1** | TCTGGTAGCAGAGACCCCAG | 688 |
|  | GGTGCAGTGCTGGGAGAG |  |
| **5_2** | ACTTCATTCCTCTGATGGGC | 670 |
|  | AGAAAGCAGAGCCAGTGGTG |  |
| **5_3** | GCCTAGGCCTCTAGGGTGAC | 733 |
|  | GGCTCAGAGTCAGAGTTCATTTG |  |

**Table S1.** Primer used for SFTPA gene sequencing.

| **SFTPB Exon** | **Primer** | **Product size** |
| --- | --- | --- |
| 1 | CTGCCTAGGAGAGGGGAGGCT | 792 |
|  | CTATGCCCCAGCCCCTACCCTG |  |
| 2 | GAGCCCACCCAGCACCCTTC | 451 |
|  | AGCACTGCTTTGTGCTAGGCAT |  |
| 3-4 | CAGGCAGGAGGTGAGCTTGCAG | 639 |
|  | AGCCTCCCCCACTCATGTGTCC |  |
| 5-6 | GGTATGCGTGTGCTCCTGGGC | 618 |
|  | GGCCGGCCTGAATAGGGGTG |  |
| 7-8 | GCCTTGAACGGGCCCTGACC | 624 |
|  | AGCTGGGTGCTGGGCAGAGA |  |
| 9-10 | GGGAGCAGAAGGGCCTCCCAT | 802 |
|  | AGGCCAGGACCACACGACAGGA |  |

**Table S2.** Primer used for SFTPB gene sequencing.

**Plasmids**

The pEYFP-*hABCA3-WT* vector with YFP fused to the C-terminus of ABCA3 was kindly provided by Prof. A. Holzinger. The ABCA3-G964D point mutation was introduced in the pEYFP-*hABCA3-WT* vector using the QuickChange Site-directed mutagenesis kit (Stratagene) with the following primers: G964D-for 5’-*GCGAGTACGACAGAACCGTCGTG*-3’ and G964D-rev 5’-*CACGACGGTTCTGTCGTACTCGC*-3’.

**Immunefluorescence**

YFP is a yellow fluorescent protein which does not need to be stained. Therefore, only LAMP3 and calnexin were stained according to the material and methods.

**Western immunoblot**

YFP was detected with a mouse-anti-GFP antibody (Clontech) and the Novex WesternBreeze Immunodetection Kit (Invitrogen). All other antibodies and procedures were as described in the materials and methods.

Acknowledgement: The pEYFP-*hABCA3-WT* vector was kindly provided by Prof. A. Holzinger.

**Supplementary Figures**


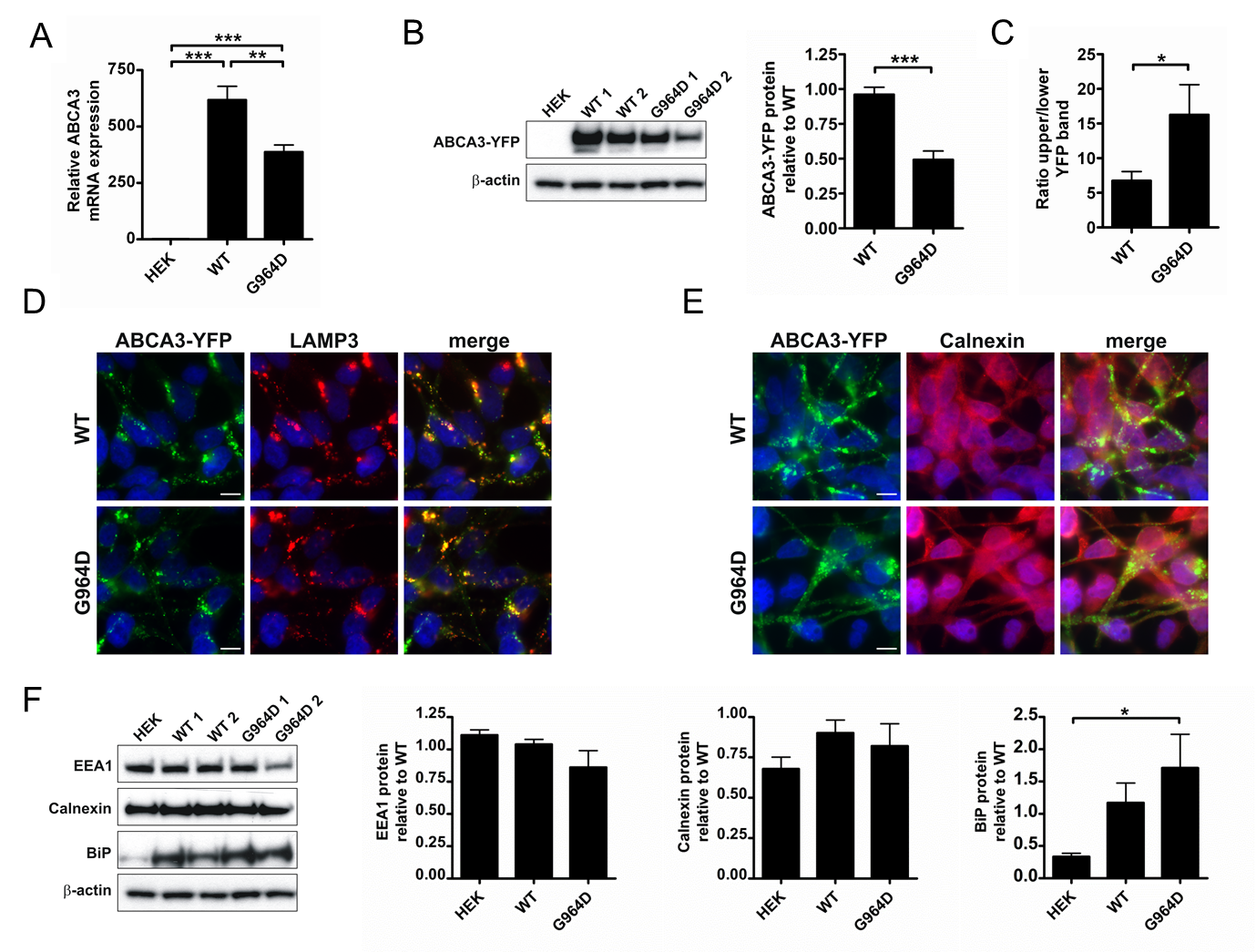


**Figure S1. Cellular effects of stable ABCA3 expression in HEK cells.**

A. ABCA3 mRNA expression levels analyzed by quantitative real time PCR. B. Western immunoblot analysis of YFP-tagged ABCA3 in total cell lysates. β-actin was used as a loading control. C. Ratio of the upper/lower ABCA3 processing form. D.Fluorescence of YFP-tagged ABCA3 and immunostaining of the lysosomal (lamellar body) marker LAMP3. E. Fluorescence of YFP-tagged ABCA3 and immunostaining of the ER marker calnexin. F. ER (calnexin, BiP) and early endosome markers (EEA1) in cells stably transfected with ABCA3. β-actin was used as a loading control. Scale bars: 7.5 µm, **P*<0.05, ***P*<0.01, ****P*<0.001.


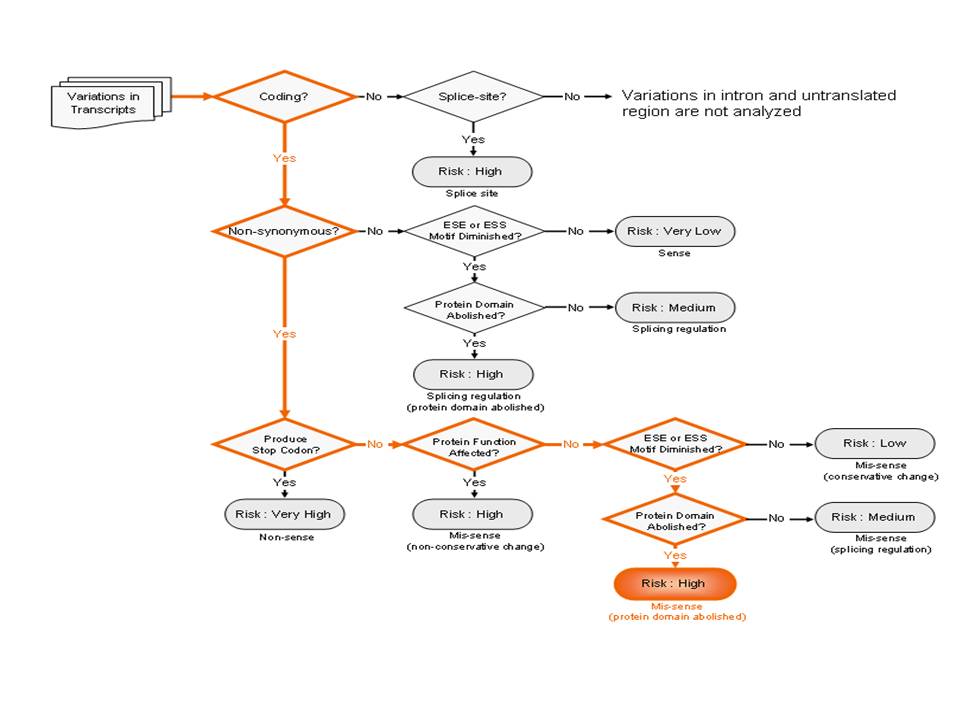


**Figure S2. Bioinformatic analysis.** Bioinformatic analysis of the ABCA3 G>A transition at nucleotide 2891 by FANS (Functional analysis of novel SNPs and mutations in human and mouse genomes) [12].
